# LHX9, a p53-binding protein, inhibits the progression of glioma by suppressing glycolysis

**DOI:** 10.18632/aging.203436

**Published:** 2021-09-17

**Authors:** Xiangying Luo, Jianwei Ge, Tao Chen, Jinfang Liu, Ziyuan Liu, Changlong Bi, Song Lan

**Affiliations:** 1 Department of Neurosurgery, XiangYa Hospital of Central South University, Changsha 410078, P.R. China; 2 Department of Neurosurgery, Renji Hospital, School of Medicine, Shanghai Jiaotong University, Shanghai 200127, P.R. China; 3 Department of Neurology, The First Affiliated Hospital of Guangzhou Medical University, Guangzhou 510120, P.R. China

**Keywords:** LHX9, p53, glioma, glycolysis, PGK1

## Abstract

Purpose: LHX9 methylation has been reported in many tumors, but its functions and related mechanisms in glioma are still unknown and need to be verified.

Methods: The protein level of LHX9 in glioma tissues was examined using western blotting and immunohistochemistry, and the functions of LHX9 in glioma cell lines were investigated using MTT and colony formation assays. In addition, the interaction between LHX9 and P53 was analyzed by immunoprecipitation, and the roles of LHX9 in cancer metabolism were explored by measuring metabolites.

Results: In this study, we found that the LHX9 expression level was decreased in glioma specimens, and the upregulation of LHX9 expression inhibited the growth of glioma cells in liquid medium and on soft agar. Regarding the molecular mechanism, we found that LHX9 interacted with p53, and downregulation of LHX9 promoted the expression of the glycolysis-related enzyme PGK1 and increased the lactic acid content. By interfering with the expression of LHX9, the tumorigenicity of glioma cells was promoted, an outcome blocked by further interference with PGK1 expression.

Conclusion: In summary, the decreased expression of LHX9 in gliomas activates the expression of the glycolysis-related enzyme PGK1, thereby promoting the development of gliomas, suggesting that the LHX9-PGK1 signaling axis can be used as a target for the treatment of glioma.

## INTRODUCTION

In recent years, an increasing number of studies have shown that abnormally active glycolytic metabolism can significantly promote the proliferation and invasion abilities of tumor cells [1]. As the most common malignant tumor in the skull, glioma has glycolytic activity in tissues that is three-fold higher than that in normal brain tissues [2, 3]. However, the molecular basis and exact mechanism of this glycolysis remodeling remain to be investigated.

Phosphoglycerate kinase 1 (PGK1), an important rate-limiting enzyme in glycolysis, can catalyze the conversion of 3-phosphoglycerate to 3-phosphoglycerate phosphate [4]. In addition to cell metabolism regulation, PGK1 is involved in a variety of biological activities, including angiogenesis, autophagy and DNA repair [5–7]. With multiple functions, PGK1 is involved in very complicated mechanisms in the occurrence and development of tumors [8]. PGK1 is highly expressed and promotes the proliferation of tumor cells [9]. PGK1 is also associated with radiochemotherapy resistance and poor prognosis [8]. Therefore, it is of great significance to investigate the regulatory mechanism of PGK1.

LHX9, LIM homeodomain (LIM-hd) transcription factor 9, is highly expressed in mesenchymal cells of the testis and ovary and plays important roles in determining the sex of animals and the differentiation of sex organs [10–13]. Valentina et al. found that LHX9 was also expressed in mouse embryonic brain tissues, especially in the diencephalon, the telencephalon, vesicles and the dorsal mesencephalon [13]. LHX9 consists of a linker region and two highly conserved cysteine-rich zinc finger structures that link to proteins [14]. According to their expression pattern and structural characteristics, LHX9-encoded transcription factors are very likely to control the differentiation fate of a variety of nerve cells [14–17].

Studies have shown LHX9 methylation in approximately 88% of high-grade gliomas and approximately 29% of nondiffuse fibroblastic astrogliomas [18]. In glioma tissues, LHX9 expression levels are significantly reduced [18]. Previous studies have shown that the expression of LHX9 significantly inhibits the migration and invasion of glioma cells [18]. However, other functions and mechanisms of LHX9 in glioma cells are still unknown.

In this study, we investigated novel functions of LHX9 in gliomas and LHX9 regulation of PGK1 and glycolysis.

## RESULTS

### Downregulation of LHX9 is associated with poor survival of glioma patients

**Figure 1:**
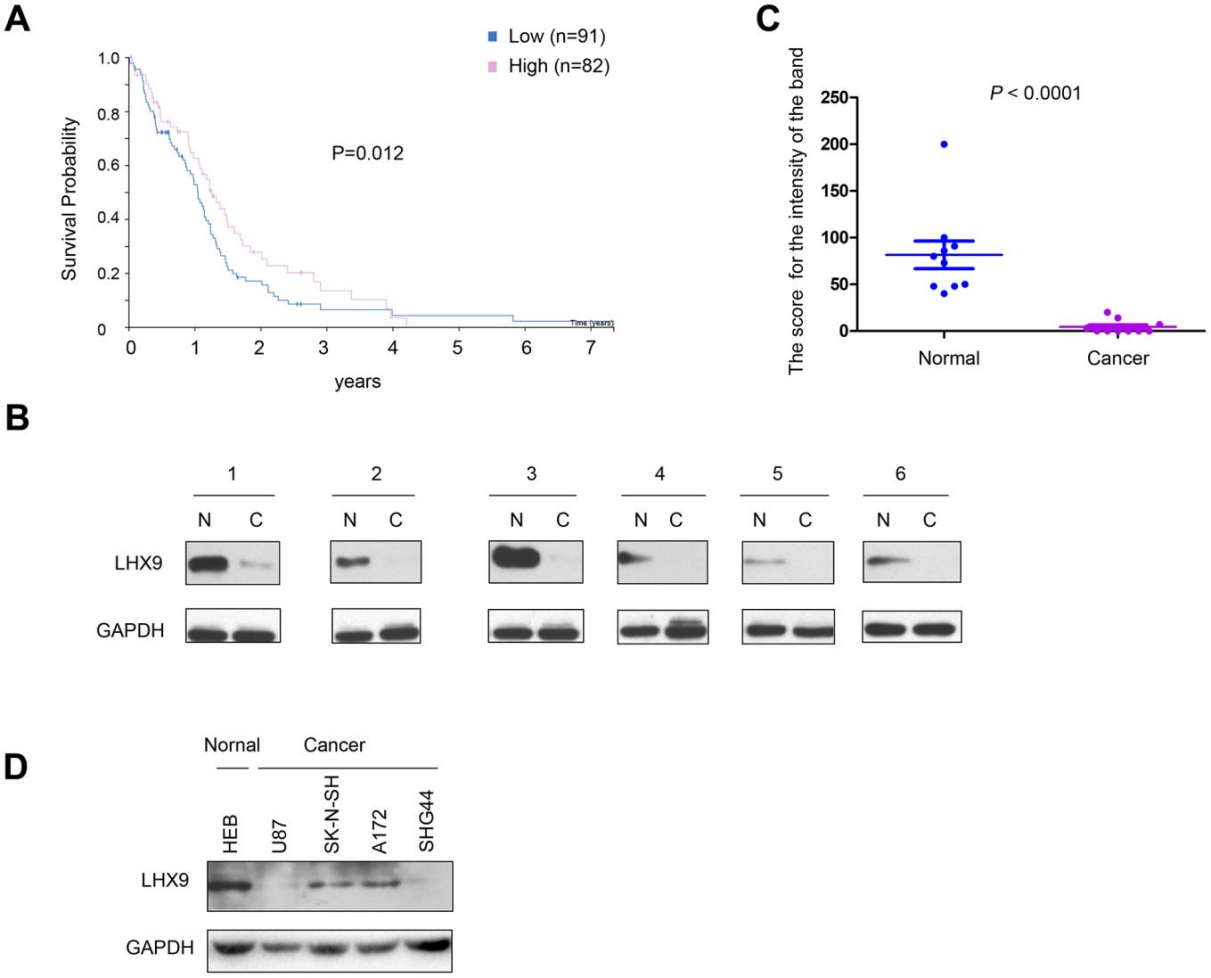
**Down-regulation of LHX9 is associated with poorer survival of glioma patients.** (**A**) GEPIA database analysis showed that LHX9 was positively correlated with survival time of patients. Patients with higher LHX9 expression levels survived better. (**B**, **C**) The expression levels of LHX9 in normal and glioma tissues were detected by Western blot, and quantified. (**D**) The expression levels of LHX9 in normal cells HEB and glioma cells were detected by Western blot.

To investigate the correlation between the expression pattern of LHX9 and the survival of glioma patients, we first searched public databases and analyzed the expression of LHX9 in glioma tissues (http://www.proteinatlas.org/ENSG00000143355-LHX9/pathology/glioma#ihc). As shown in Figure 1A, patients with high expression of LHX9 had a better prognosis. Then, we measured the expression of LHX9 in glioma tissues and paracarcinoma tissues. The experimental results showed negligible protein expression of LHX9 in most glioma tissues (Figure 1B, 1C). Consistent with this result, LHX9 was expressed at high levels in HEB normal human brain glial cells and at low levels in glioma cells (U87, SK-N-SH, A172, and SHG44 cells) (Figure 1D). These results suggest that LHX9 expression was downregulated in the gliomas.

### LHX9 inhibited the growth and colony formation of glioma cells

**Figure 2:**
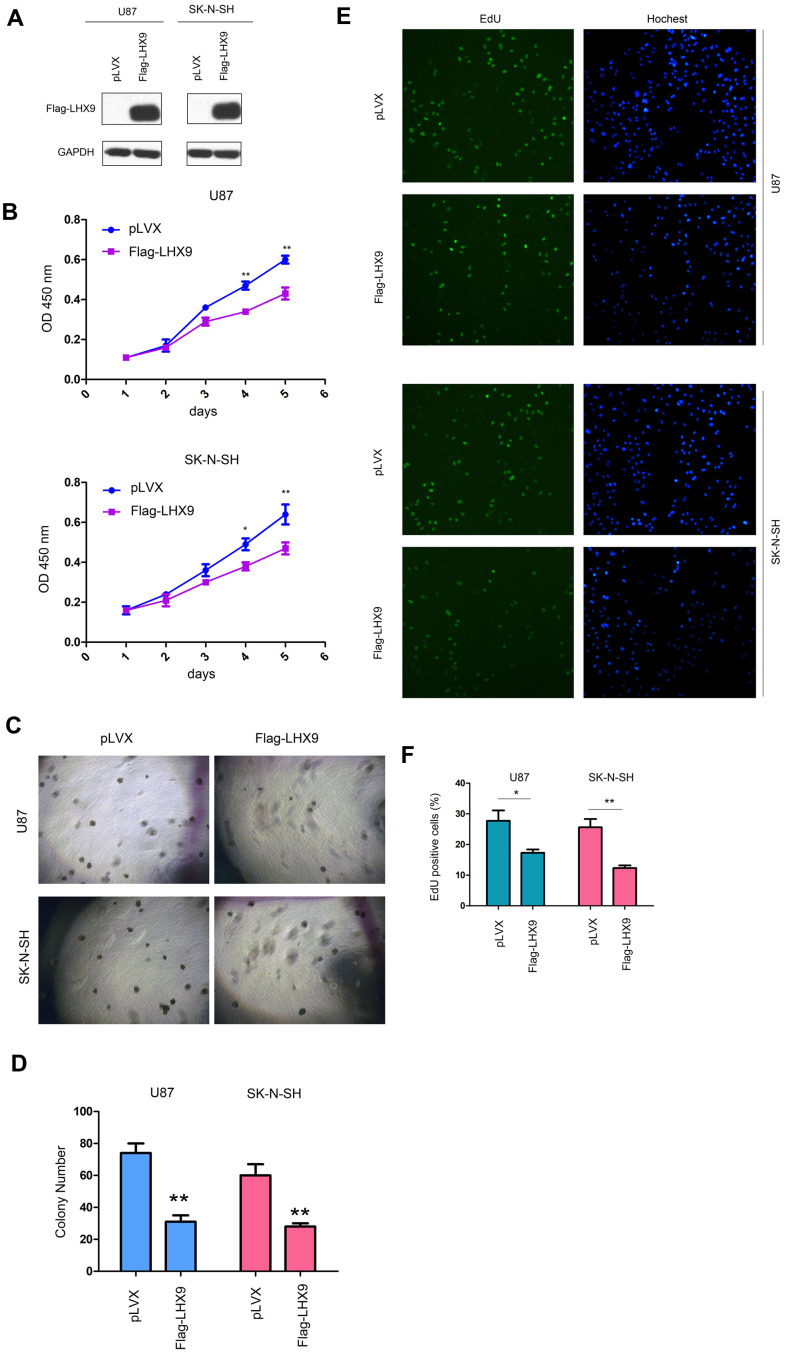
**LHX9 overexpression inhibited the growth of glioma cells.** (**A**) Overexpression of Flag-labeled LHX9 in U87 and SK-N-SH cells. Flag-LHX9 expression plasmid was transfected into U87 and SK-N-SH cells using lipofectamine 2000. Cells were screened with puromycin for 1 week, and then Flag-LHX9 expression was identified. (**B**) The effect of LHX9 expression on the growth of U87 and SK-N-SH cells was detected by CCK8 assay. (**C**, **D**) The effect of LHX9 expression on the anchorage-independent growth of U87 and SK-N-SH cells was detected by soft agar assay. (**E**, **F**) The EdU assay was performed. Details about the EdU assay were described in the “Materials and methods”. *, *P*<0.05; **, *P*<0.01.

To investigate the functions of LHX9 in glioma cells, we overexpressed flag-tagged LHX9 (Flag-LHX9) in glioma cells (Figure 2A) and measured the effect of LHX9 expression on the growth of glioma cells using MMT and soft agar assays. The results showed that upregulating the expression of LHX9 in glioma cells not only inhibited the growth of cells in liquid medium (Figure 2B) but also inhibited the anchorage-independent growth of glioma cells (Figure 2C, 2D). To confirm the roles of LHX9 in the proliferation of glioma cells, an EdU assay was performed. As shown in Figure 2E, 2F, overexpression of LHX9 decreased the percentage of EdU-positive cells (Figure 2E, 2F).

**Figure 3:**
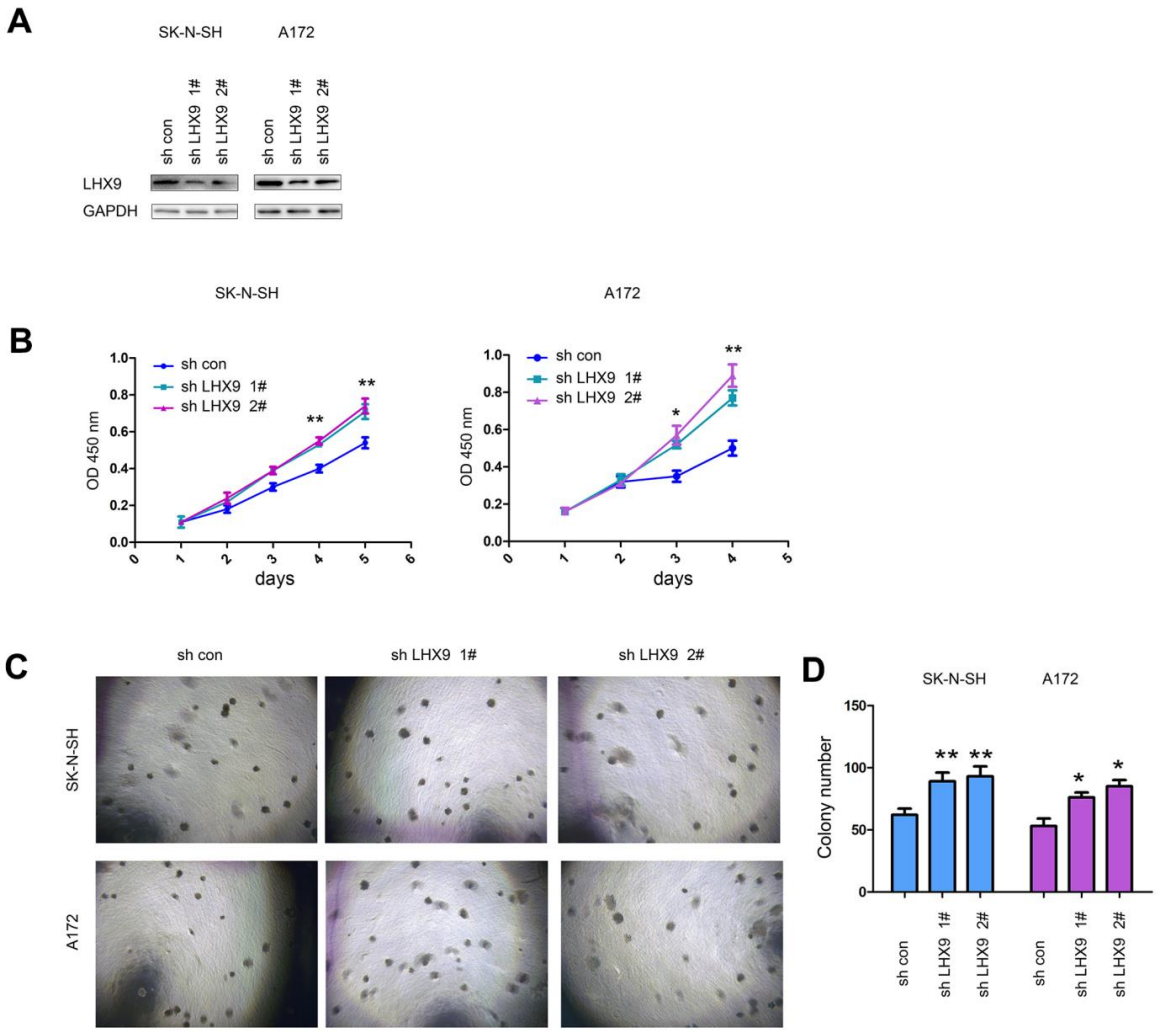
**Interfering with LHX9 expression accelerated the growth of glioma cells.** (**A**) Interference with LHX9 expression in A172 and SK-N-SH cells. The target sequence was cloned into the pL.KO.1 vector, the virus was packaged to infect A172 and SK-N-SH cells. Cells were screened with puromycin for 1 week, and then the expression of LHX9 was identified. (**B**) The effect of interference with LHX9 expression on the growth of A172 and SK-N-SH cells was detected by CCK8 assay. (**C**, **D**) The effect of interference with LHX9 expression on the anchorage-independent growth of A172 and SK-N-SH cells was detected by soft agar assays. *, *P*<0.05; **, *P*<0.01.

In addition, we investigated the biological functions of endogenously expressed LHX9 in gliomas. We knocked down the expression of LHX9 in SK-N-SH and A172 glioma cells (Figure 3A). The experimental results showed that downregulating the expression of LHX9 in SK-N-SH and A172 glioma cells accelerated the growth of the cells in liquid medium (Figure 3B) and colony formation on soft agar (Figure 3C, 3D).

### Interaction of LHX9 and p53

**Figure 4:**
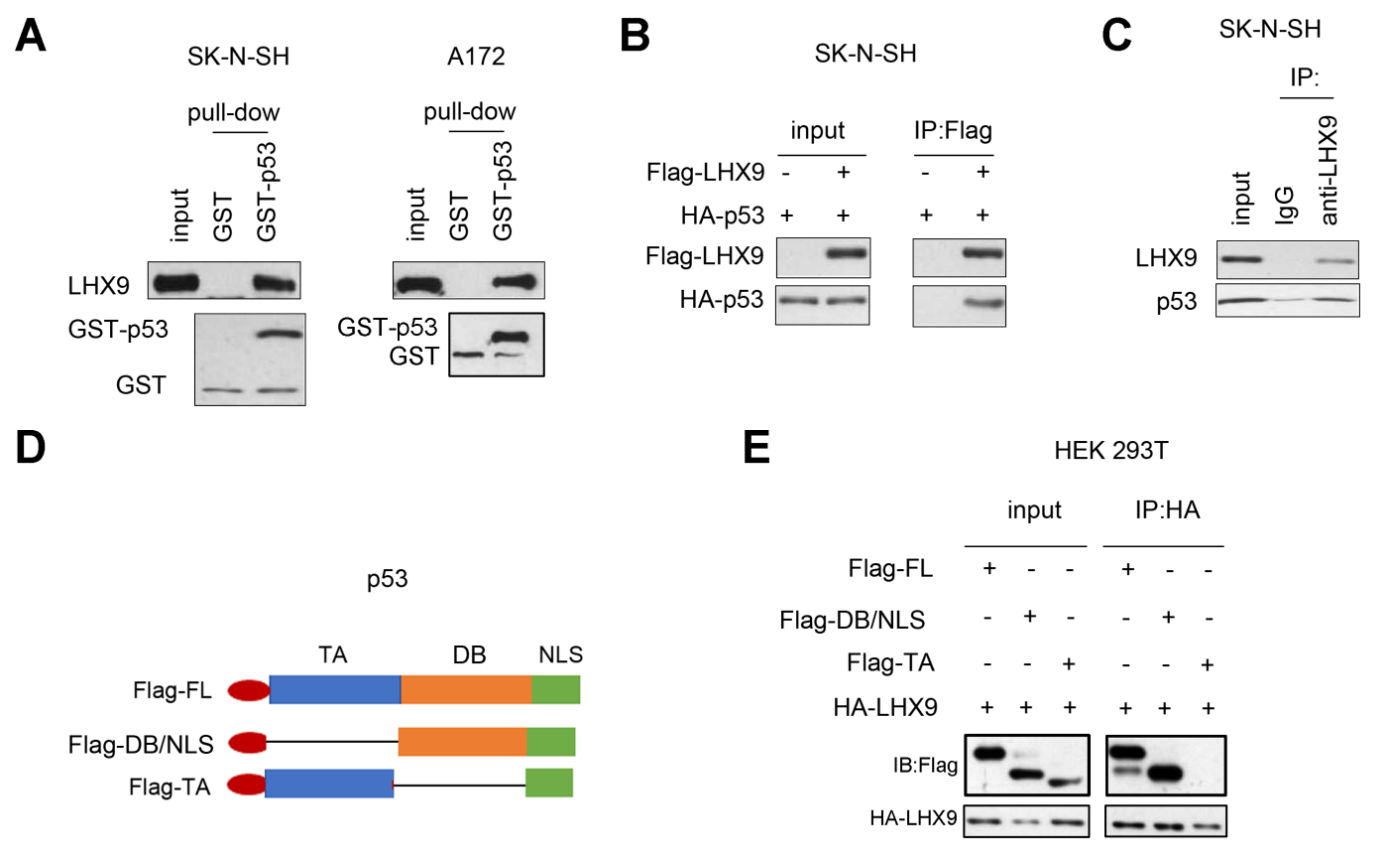
**Interaction between LHX 9 and p53.** (**A**) The interaction between LHX9 and fusion protein GST-p53 was detected by GST pull-down assay. 10 μg of GST-p53 fusion protein incubated with SK-N-SH and A172 cell lysis. (**B**) The interaction between the exogenously expressed Flag-LHX9 and HA-p53 was detected by co-immunoprecipitation assay. (**C**) The interaction between endogenously expressed LHX9 and p53 was detected by co-immunoprecipitation assay. (**D**) Schematic diagram of different truncated mutants of p53. (**E**) The domain of p53 that interacted with LHX9 was identified by co-immunoprecipitation.

To investigate the mechanism by which LHX9 regulates the growth and colony formation of glioma cells, we analyzed the interactions between LHX9 and a series of proteins involved in cell growth regulation. Figure 4A shows that the fusion protein GST-p53 interacted with endogenously expressed LHX9, as determined by the GST pull-down assay. In addition, coimmunoprecipitation assays showed that p53 and LHX9 formed a complex (Figure 4B, 4C). Subsequently, we constructed a series of p53 truncated mutants (Figure 4D), and these mutants were cotransfected with LHX9 into HEK293T cells. The experimental results showed that the DB (DNA binding) domain of p53 mediated its interaction with LHX9 (Figure 4E).

### Downregulation of LHX9 expression activated glycolysis in tumor cells

**Figure 5:**
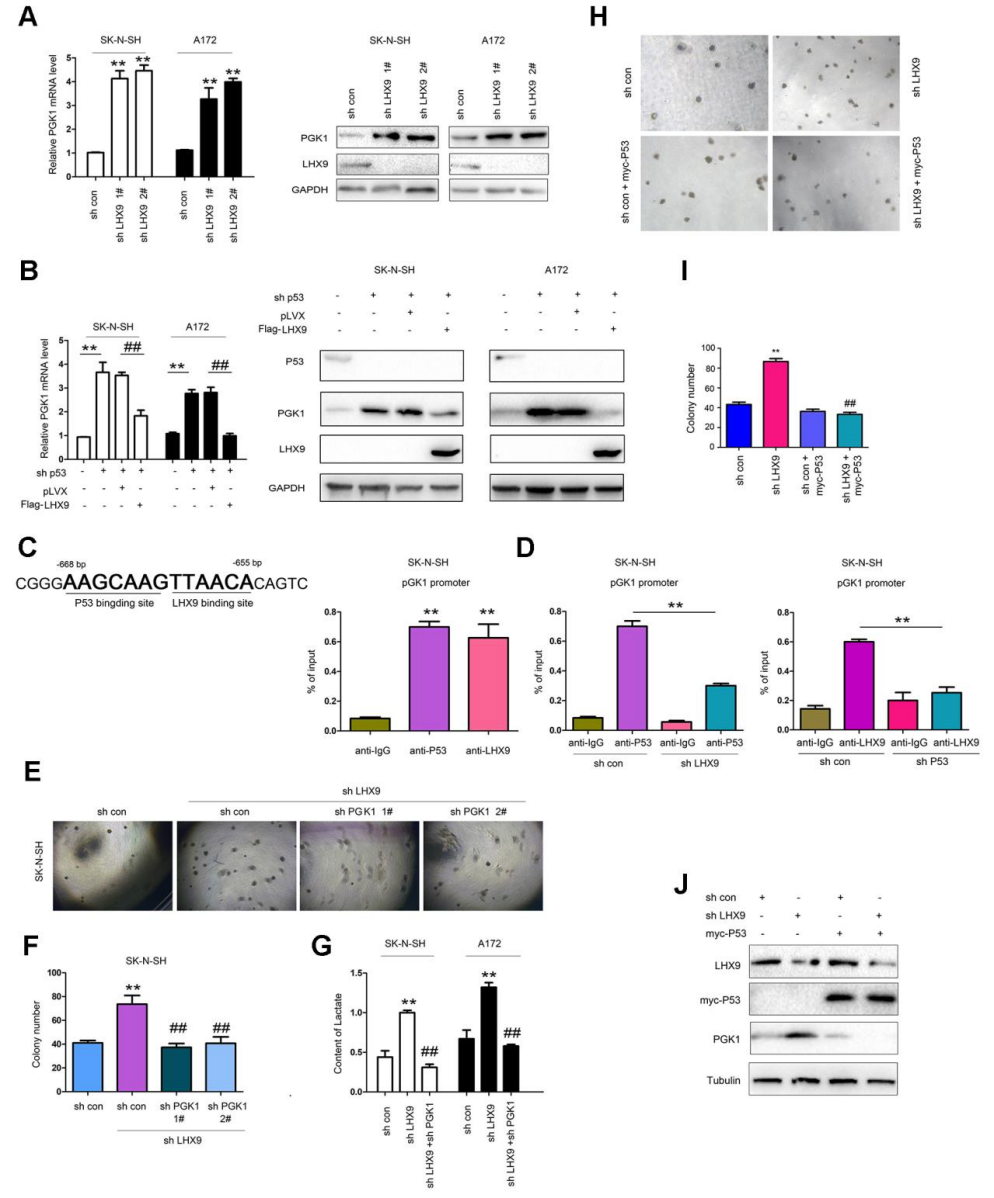
**LHX9 inhibited glycolysis by down-regulating PGK1 expression.** (**A**) Interfering with LHX9 expression up-regulated the mRNA level of PGK1. (**B**) Overexpression of LHX9 inhibited the induction of PGK1 by knockdown of p53. (**C**) Left: the schematic illustration of the PKG1 promoter with labeled p53 and LHX9 binding sites. Right: Chromosomal immunoprecipitation assay demonstrated that p53 and LHX9 bound to the PGK1 promoter. (**D**) Chromosomal immunoprecipitation assay demonstrated that down-regulating LHX9 expression inhibited the binding of p53 to the PGK1 promoter, and vice versa. (**E**, **F**) Soft agar assay demonstrated that down-regulating the expression of PGK1 abolished the increase in colony formation caused by down-regulation of LHX9. (**G**) Interfering with LHX9 expression increased the lactic acid content. This increase could be suppressed by interfering with PGK1. (**H**, **I**) The effects of P53 restoration on the anchorage-independent growth of SK-N-SH were examined. (**J**) The effects of P53 restoration on the PGK1 expression of SK-N-SH were examined. ##, *P*<0.01; **, *P*<0.01.

P53 suppresses glycolysis of tumor cells. Considering the interaction between LHX9 and p53, we first investigated the effect of LHX9 on glycolysis in glioma cells. As shown in Figure 5A, hindered LHX9 expression led to the upregulated expression of PGK1. Knockdown of p53 induced the expression of PGK1, which was abolished by the overexpression of LHX9 (wild-type p53 in SK-N-SH cells and A172 cells) (Figure 5B). In the screening of the PGK1 promoter, one-half of the P53-binding site sequence (AAGCAAG) was adjacent to the LHX9-binding site sequence (TTAACA) in the region from -655 bp to -668 bp (Figure 5C, left). This finding suggests the binding of P53 and LHX9 to the PGK1 promoter. Chromosome immunoprecipitation (ChIP) assays showed that p53 and LHX9 were bound to the PGK1 promoter at the same time (Figure 5C, right). However, interfering with LHX9 expression inhibited the binding of p53 to the PGK1 promoter, and vice versa (Figure 5D). Next, we investigated whether PGK1 mediated the biological functions of LHX9. In SK-N-SH cells with dampened LHX9 expression, interference of PGK1 expression restored the colony formation induced by the downregulation of LHX9 (Figure 5E, 5F). Moreover, interfering with the expression of LHX9 increased the lactic acid content, while interfering with the expression of PGK1 restored the lactic acid content (Figure 5G). To determine whether the promoting effects of LHX9 on the anchorage-independent growth of glioma cells were dependent on P53, we restored the expression of P53 in LHX9-knockdown cells. As shown in Figure 5H–5J, the restoration of P53 abolished the advantages of anchorage-independent growth and PGK1 expression caused by LHX9 knockdown.

### Interfering with LHX9 expression promoted the tumorigenicity of glioma cells

**Figure 6:**
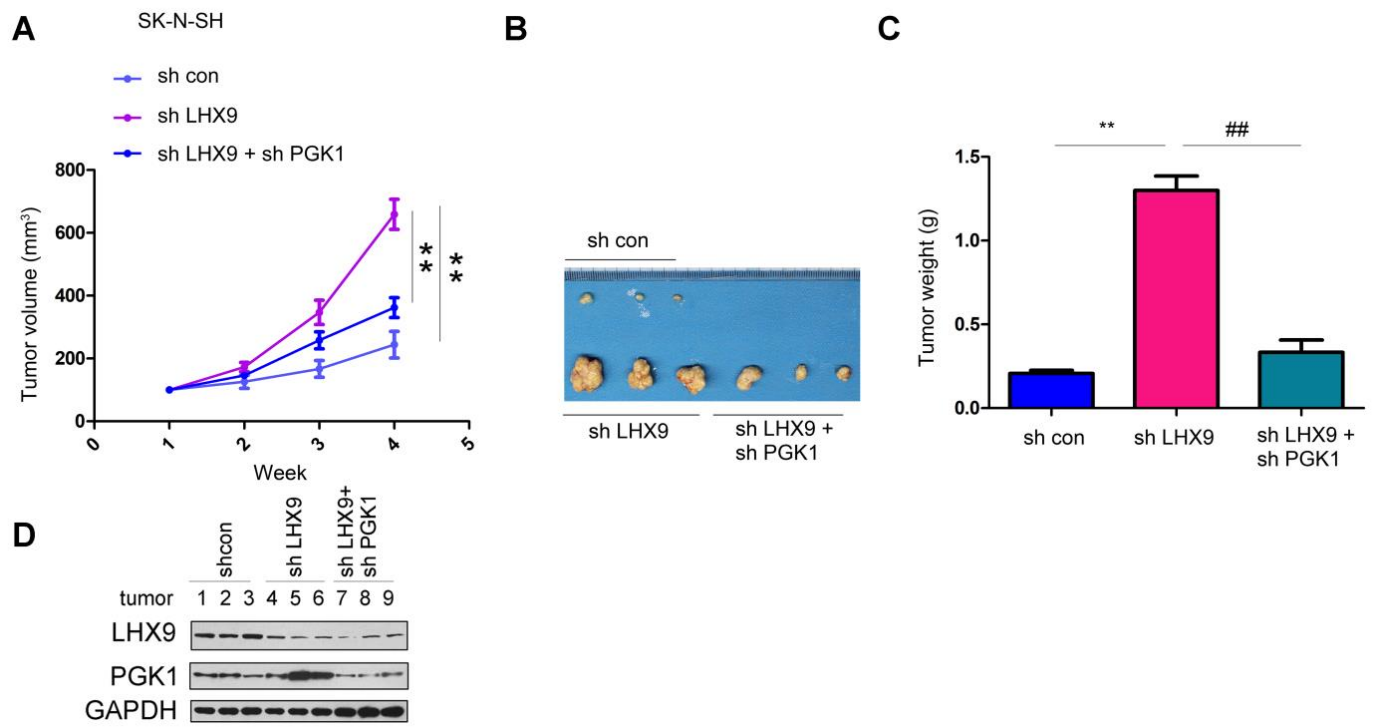
**LHX9 down-regulation accelerated the *in vivo* tumorigenicity of glioma cells.** (**A**) Tumor growth curve. SK-N-SH cells were interfered with the expression of LHX9 or the expressions of both LHX9 and PGK1. The *in-vivo* tumor formation experiments were performed in nude mice and the tumor volume was recorded. (**B**, **C**) Tumor morphology and tumor weight. (**D**) Expressions of LHX9 and PGK1 in tumors were detected by Western blot. ##, *P*<0.01; **, *P*<0.01.

Interfering with LHX9 promoted the growth of SK-N-SH cells in nude mice. Interfering with PGK1 restored the tumor volume and weight caused by downregulation of LHX9 expression (Figure 6A–6C). We extracted proteins from the formed tumors to detect the expression of LHX9 and PGK1 (Figure 6D).

## DISCUSSION

Gliomas are among the common malignancies in the skull [19]. Patients with gliomas have an extremely poor prognosis, and the need for new treatment methods is urgent [19]. Therefore, it is of significance to the treatment of glioma to study the molecular mechanism of glioma progression. Glycolysis is a basic feature of tumor cell metabolism [20, 21]. An in-depth analysis of the regulatory mechanism of glycolysis in glioma cells is expected to provide potential targets for the treatment of gliomas. In this study, we found that the transcription factor LHX9 was downregulated in gliomas. LHX9 interacted with p53 to inhibit the expression of PGK1, thereby inhibiting the progression of gliomas. This study suggests that PGK1 is very likely to be an important therapeutic target for gliomas.

An important finding of this study is that LHX9 overexpression inhibits the colony-forming ability of glioma cells on soft agar. Although Vladimirova V.et al. found that LHX9 had no effect on the growth of glioma cells, their observation time window was 72 hours [18]. In our CCK-8 assay, we also observed that LHX9 expression had little effect on the growth of glioma cells in the first 72 hours, but within a time window of 96 hours (4 days), we observed that LHX9 had inhibitory effects on cell growth and that downregulation of LHX9 expression promoted the growth of glioma cells. In addition, we also observed that LHX9 inhibited the colony formation of glioma cells on soft agar, suggesting that LHX9 inhibited the anchorage-independent growth of glioma cells. None of these findings were not observed by Vladimirova V et al. [18].

Another important finding of this study is that LHX9 inhibits PGK1 expression by interacting with p53. LHX9 interacts with the p53 DNA-binding domain, suggesting that it very likely regulates the transcriptional activity of p53. In tumor formation experiments, we observed that interference with downstream PGK1 expression almost completely reversed the tumorigenicity caused by the downregulation of LHX9 expression, suggesting that treatment with inhibitors of PGK1 is highly likely to benefit patients with glioma. Consistent with this finding, PGK1 has been found to be upregulated in various tumors and to promote tumor progression.

In conclusion, we have revealed the molecular mechanism of glioma inhibition by LHX9 in this study. The LHX9/p53-PGK1 signaling pathway is likely to be an important target for the treatment of glioma.

## MATERIALS AND METHODS

### Cell culture and transfection

Glioma cells (U87, SK-N-SH, SHG44 and A172 cells) and normal HEB cells were purchased from Shanghai Cell Bank, Chinese Academy of Sciences. The cells were cultured in DMEM containing 10% serum and antibiotics.

Cells were cultured in an incubator at 37° C with 5% CO_2_. The cells were transfected using Lipofectamine 2000 following the manufacturer’s instructions. Screening was performed using 1 μg/ml puromycin 48 hours after cell transfection. Seven days later, the surviving cells were mixed, and gene expression was determined by Western blot analysis.

### Clinical specimens

Clinical specimens were collected from the Department of Neurosurgery, XiangYa Hospital of Central South University, and informed consent was obtained from the patients from whom the specimens were collected. The specimens were fixed with formaldehyde, dehydrated with ethanol, embedded in paraffin, and cut into 5-μm sections for subsequent analysis.

### Western blot analysis

After washing twice with PBS, cells were lysed with RIPA buffer (1% NP-40, 0.1% SDS, 0.5% deoxycholate, 50 mM Tris [pH 7.4], and a protease inhibitor cocktail) on ice for 15 min, scraped with a scraper, and centrifuged at 12,000 rpm for 15 min at 4° C. After measuring the protein concentration with Bradford reagent (Sigma), the protein samples were adjusted, 6× loading buffer was added, and the samples were boiled for 5 min. Then, electrophoresis was performed. Then, the proteins were transferred to a polyvinylidene difluoride membrane (Millipore). After incubation with TBST containing 5% skimmed milk (25 mM Tris, 150 mM NaCl, 0.05% Tween 20 [pH 7.5]) for 1 hour at room temperature, the primary antibody was incubated overnight with the membrane at 4° C. After washing the membrane three times with TBST, a horseradish peroxidase-conjugated secondary antibody was added, and the membrane was incubated for 1 hour at room temperature. The membrane was washed three times with TBST and developed with enhanced chemiluminescence (Pierce). Antibodies against GST (10000-0-AP), GAPDH (10494-1-AP), HA (51064-2-AP) and Flag (20543-1-AP) were obtained from Proteintech, and antibodies against LHX9 (ab224357), P53 (ab26) and PGK1 (ab199438) were obtained from Abcam.

### Cell growth assay

One hundred microliters of cell suspension (containing 1000 cells) was inoculated into wells of a 96-well plate, and the time was recorded as “day 0”. Ten microliters of CCK-8 solution was added to each well on days 1, 2, 3, and 4 and then incubated in an incubator for 4 hours. The absorbance at 450 nm was measured with a microplate reader.

### Edu assay

Cells were plated into a 96-well plate (20000 cells/well). Cell proliferation was evaluated using a Cell-Light EdU Apollo 567 *in vitro* kit (RiboBio, C10310-1). A fluorescence microscope was used to acquire images for analysis. The percentage of positively stained cells was calculated.

### Anchorage-independent growth assay

A soft agar assay was performed with 12-well plates. The soft agar consisted of two layers: the upper layer and the lower layer. The lower layer of agar was mainly used to embed the 12-well plates; the agar concentration was 0.5%, and the serum concentration was 10%. The upper agar was used for resuspending the cells; the agar concentration was 0.35%, and the serum concentration was 10%. An inhibitor was mixed in the upper agar. First, the agar was heated to 37° C, and the lower layer of agar was paved. After agar coagulation, 2,000 cells were added to the upper layer of agar, and after mixing well, cells were seeded on the lower layer of agar. The cells were photographed and counted after incubation for 14 days at 37° C.

### Knockdown of LHX9 and PGK1 expression

Retroviruses that interfere with LHX9 expression were purchased from Shanghai GeneChem Biotechnology Co., Ltd. The viruses were removed after 8 hours of incubation with cell culture. Twenty-four hours later, puromycin (1 μg/ml) was added for screening. After seven days, surviving cells were collected, and the expression of LHX9 was verified.

### GST pull-down assay

Cells were collected, lysed and centrifuged at 12,000 rpm for 20 min at 4° C, and then, the supernatant was collected. The supernatant was incubated with 10 μg of GST or GST-p53 fusion protein at 4° C overnight, and then, Sepharose 4B GST gel beads were added and incubated for another 4 hours. The gel beads were washed 3 times with PBST buffer for 5 min each time. Then, 30 μL of loading buffer was added and boiled for 5 min at 100° C, and the supernatant was collected for Western blotting.

### Coimmunoprecipitation

SK-N-SH and A172 cells were collected, lysed and centrifuged at 12,000 rpm for 20 min at 4° C, and the supernatant was collected. The supernatant was mixed with the primary antibody and incubated overnight at 4° C. Then, protein A gel beads were added and incubated for another 4 hours. The gel beads were washed 3 times with PBST buffer for 5 min each time. Then, 30 μL of loading buffer was added and boiled for 5 min at 100° C. The supernatant was used in Western blot analysis.

### qPCR

For qPCR, 20 μl of Hieff qPCR SYBR® Green Master Mix (No Rox Plus) was used for the amplification reaction. The system included 10 μL of PCR MIX, 0.4 μL of 10 μm forward primer, 0.4 μL of 10 μm reverse primer, and 3 μL of cDNA template and supplemented to 20 μL with ddH_2_O. All reactions were performed in duplicate and detected with a Thermo Scientific™ PikoReal™ Real-Time PCR detection system. The reaction conditions were as follows: 95° C for 3 min; 40 cycles of 94° C for 30 s and 60° C for 30 s; and finally, 95° C for 15 s, 60° C for 60 s, and 95° C for 15 s. The melt curve was plotted to determine the specificity of the amplification. The forward primer of PGK1 was 5’-agataacaaacaaccagagg-3’, and the reverse primer was 5’-acagacccagcagctgggtt-3’.

### ChIP

ChIP assays were performed by using a ChIP kit from Cell Signaling Technology. Antibodies against p53 and LHX9 were used for ChIP. The IP results with normal IgG or specific antibody were then used to calculate the relative nonspecific background and specific occupancy. The ChIP primers were F, 5’-GATGTAATTTTTCAATGG-3’, and R, 5’-TAACTGCCAAGATGTAAC-3’.

### Determination of lactic acid content

Cells were cultured in Dulbecco’s modified Eagle’s medium without phenol red for 15 hours. Then, the culture medium was harvested to measure the lactate concentrations. Lactate levels were quantified using a lactate assay kit (BioVision, CA). All values were normalized to the relative protein levels measured using a bicinchoninic acid (BCA) protein assay.

### Subcutaneous tumor formation experiments

Four-week-old male nude mice were assigned to 3 group, with 3 mice in each group. One group of mice was injected with sh control SK-N-SH cells (1*10^6^ cells/injection), one group of mice was injected with sh LHX9 SK-N-SH cells (1 * 10^6^ cells/injection), and the other group was injected with sh LHX9+sh PGK1 SK-N-SH cells (1*10^6^ cells/injection). The mice were sacrificed 4 weeks after injection, tumor tissues were removed and weighed, and the expression of LHX9 and PGK1 was measured.

### Ethics approval and consent to participate

Ethical approval Research involving animals: All applicable international, national, and/or institutional guidelines for the care and use of animals were followed.

Research Involving human participants: All procedures performed in studies involving human participants were in accordance with the ethical standards of the institutional and/or national research committee and with the 1964 Helsinki declaration and its later amendments or comparable ethical standards.

### Consent for publication

Written informed consent for publication was obtained from all the authors.

### Availability of data and materials

The data that support the findings of the study are available from the corresponding author upon reasonable request.
